# Relationship between objectively measured intensity of physical activity and self-reported enjoyment of physical activity

**DOI:** 10.1016/j.pmedr.2017.06.004

**Published:** 2017-06-09

**Authors:** Thea Schwaneberg, Franziska Weymar, Sabina Ulbricht, Marcus Dörr, Wolfgang Hoffmann, Neeltje van den Berg

**Affiliations:** a Institute for Community Medicine, Section Epidemiology of Health Care and Community Health, University Medicine Greifswald, Greifswald, Germany; b DZHK (German Centre for Cardiovascular Research), Partner Site Greifswald, Germany; c Institute of Social Medicine and Prevention, University Medicine Greifswald, Greifswald, Germany; d Department of Internal Medicine B, University Medicine Greifswald, Greifswald, Germany

**Keywords:** Physical activity, Middle-aged people, Prevention, Cardiovascular diseases, Enjoyment scale

## Abstract

Physical activity is an important factor for the maintenance of health. Enjoyment of physical activity is essential to motivate persons to engage in sufficient physical activity. We examined whether self-reported enjoyment of PA is associated with objective measurement of the intensity of PA.

A cardiovascular examination program was provided for individuals aged 40–75 years without a history of cardiovascular events in Greifswald, Germany between 2012 and 2013. Participants (n = 255) were asked to wear a three-axial accelerometer device (ActiGraph, GT3X +, Pensacola, Florida, USA) for 7 consecutive days. After wearing the device, the participants were asked to complete the 18-item self-administered physical activity enjoyment scale (PACES). Participants' (n = 200) daily minutes of moderate-to-vigorous physical activity (MVPA) and their enjoyment of PA were analysed in a linear regression approach.

The mean age of the participants was 56.3 ± 9.7 years, 41.0% were male. The average MVPA duration was 44.4 ± 27.3 min per day. In the regression analysis, enjoyment of PA was positively associated with MVPA (β = 0.18, 95% CI (0.05; 0.31), p = 0.009), participants with higher enjoyment of PA showed higher MVPA.

We found a positive association between MVPA and enjoyment of PA, although for male participants only. Between bouted MVPA and enjoyment of PA there was no significant relationship.

## Highlights

• People with regular physical activity have a benefit regarding cardiovascular diseases.
• The World Health Organisation recommends a moderate to vigorous intensity.
• Most of time middle-aged people sit and do not enough sufficient physical activity.
• Accelerometry measured the physical activity of people in an objective way.
• Enjoyment is a useful tool to increase objectively measured physical activity.

## 1 Introduction

Physical activity (PA) is defined as “any bodily movement produced by the skeletal muscle that results in energy expenditure” (Westerterp, 1999). The international recommendations by the World Health Organisation (WHO) for PA for persons < 65 years are 150 minutes moderate or 75 minutes vigorous activity per week (WHO, 2016) and includes work-related activity, leisure time activity, but also sporadic and bouted PA, exercise training, and sports.

PA can reduce the risk for cardiovascular diseases (CVD), overweight, falls (Blair et al., 1989), obesity, diabetes type II (Kriska et al., 2003), depression (Strawbridge et al., 2002), perceived stress (Aldana et al., 1996), and fractures (Stattin et al., 2017). On the other hand, inactivity is associated with higher all-cause mortality (Ekelund et al., 2015, Lozano et al., 2012), coronary artery disease, stroke, and hypertension (Katzmarzyk et al., 2000). Compared to inactive subjects, people with sporadic and habitual PA have a benefit regarding CVD (Hu et al., 1999). Further, habitual PA can improve other factors including mental health, social contacts, self-confidence, healthy aging (Booth et al., 2000, Leslie et al., 1999), quality of life (Elavsky et al., 2005, Gill et al., 2013, Rejeski and Mihalko, 2001) and, especially, health-related quality of life (HQOL) (Bize et al., 2007).

In a meta-analysis regarding cardiovascular diseases and PA levels, the overall RR for the group with high level of leisure time PA was 0.76, 95% CI (0.70; 0.82) for men and 0.73 (95% CI 0.68; 0.78) for women. A high level of leisure time PA resulted in a protective effect regarding CVD. For moderate level of occupational PA, a similar protective effect has been observed (Li and Siegrist, 2012). But only 13% subjects are sufficiently physical active to profit of the preventive effect of PA. Furthermore, PA decreases with higher age (Mensink, 2003).

The level of intensity and time duration of the PA with the highest health benefit depends on the subjects' health status. The public health guidelines for physical activity implicate that PA should be performed in short consecutive bouts (at least 10 minute intervals) (Haskell et al., 2007, Haskell and Nelson, 2008, Tremblay et al., 2011), whereas sporadic PA could be easier and better accepted for mostly sedentary people (Murphy et al., 2009) and has a comparable positive health benefit (Robson and Janssen, 2015).

Accelerometry allows a direct measurement of PA during a given time. Direct measurements are objectively and avoid recall or response bias (Prince et al., 2008). PA is influenced by personal, social, and environmental factors (Sallis et al., 1997). One personal factor is to enjoy being physical active. Enjoyment of PA is both a predictor and a positive secondary effect of PA (Booth et al., 2000, Leslie et al., 1999). Predominantly, physical activity is measured by self-report questionnaires (Cleland et al., 2017, Loprinzi and Davis, 2016), but there is a possible recall bias on participants' side.

The objective of this study was to investigate the association between i. objectively measured MVPA per day and ii. bouted MVPA per day with self-reported enjoyment of PA in subjects aged 40–75 years without a history of CVD. The high proportion of inactive people worldwide show that effective strategies to enhance adults' physical activity level are needed to enhance their health status (Lee et al., 2012, Schneider et al., 2017). We postulate as hypothesis that self-reported enjoyment of PA has a significantly positive influence on individuals' PA level or weekly time in moderate-to-vigorous physical activity (MVPA). The time of MVPA per day and bouted (at least 10 consecutive minutes) MVPA per day was further used to extract the subjects' most intensive activities as defined in the recommendations by the WHO (2016).

## 2 Methods

### 2.1 Study population

Adults in the age range of 40–75 years were included in a cardiovascular examination program to assess risk factors and lifestyles between 2012 and 2013. The inclusion criteria were: no history of cardiovascular events (myocardial infarction, coronary intervention, and stroke), no diabetes mellitus, no multi-resistant pathogens, and no obesity ≥ grade II (body mass index < 35 kg/m^2^). The recruitment between June 2012 and December 2013 was performed in three settings: in general medical practices, in job agencies, and through an invitation letter by a statutory health insurance. All participants received a voucher about 15 Euros wearing the accelerometer for seven days.

The participants of the study were examined in the DZHK cardiovascular examination center in Greifswald, Germany. More detailed information about this study and the contents of the examination has been provided in a previous publication by Weymar et al. (2015). The description of the study sample size and randomization for the four study groups is described by van den Berg et al. (2017). Data as age, sex, education, occupation, current smoking status, general health status, and the intrinsic motivation stages of change of PA (Marcus et al., 1992) were assessed at baseline.

### 2.2 Ethical approval and trial registration

The study (identifier DRKS00010996 at the German Clinical Trial Register (www.drks.de), registered at 24th August 2016, retrospectively registered) was approved by the clinical ethical committee of the University Medicine Greifswald (protocol number BB41/12). All participants gave informed consent.

### 2.3 Accelerometer data

All participants were asked to wear a three–axial accelerometer device (GT3X + ActiGraph, Pensacola, Florida, USA), which measures the intensity, frequency, and duration of PA (Prince et al., 2008). The time period of seven consecutive days including weekdays and the weekend was used to examine both occupational and leisure time activity profiles (Caspersen et al., 1985, Strath et al., 2013) and to reduce intra-individual variance (Gretebeck and Montoye, 1992). The participants were instructed to wear the accelerometer on the right hip on an elasticized belt during all activities (expect during water activities).

The subjects were randomized in four groups to wear the accelerometer either at day and night or during daytime only, additionally with or without two supportive phone calls. This design was chosen to examine which group showed the best adherence to wearing the accelerometer (van den Berg et al., 2017). In the analysis described here, the participants of four groups were analysed together.

Accelerometer data were collected in 10 second epochs and defined as valid data if the accelerometer device was worn for at least 10 h/day for at least 4 days (Inoue et al., 2011, Roth and Mindell, 2013). Moderate physical activity (MPA) was determined between 2020 and 5998 counts per minute (Sasaki et al., 2011). Non-wearing time was calculated by the Troiano algorithm as at least 60 consecutive minutes of zero activity intensity counts, with allowance for 1–2 min of counts between 0 and 100 (Troiano et al., 2008). Vigorous physical activity (VPA) was defined as at least 5999 counts per minute (Troiano et al., 2008). Therefore, moderate-to-vigorous physical activity (MVPA) is defined as at least 2020 counts per minute and summarized MPA and VPA. Bouted MVPA is defined as MVPA for at least 10 consecutive minutes. Sedentary behaviour or light activity (up to 2019 counts per minute) was not included in the analysis.

### 2.4 Enjoyment of physical activity questionnaire

After wearing the accelerometer for seven days, the participants were asked to complete the 18-item self-report questionnaire physical activity enjoyment scale (PACES) (Kendzierski and DeCarlo, 1991). The participants were asked “how you feel at the moment about the physical activity you have been doing” (Mullen et al., 2011). This questionnaire is a validated instrument to measure the emotion enjoyment of PA and is composed of eighteen bi-polar items regarding positive feelings (enjoy, like, happy, energizing) and negative feelings (hate, dislike, depressed, frustrated) of being physical active (Jekauc et al., 2013). The items are listed bi-polar to prevent one-sided biased answering of the participants. The one-dimensional score is defined as the sum of the eighteen 7-point Likert scale items (score ranges between 18 and 126 points). A high score represents a high enjoyment of PA. Compared to the intrinsic motivation based on the five stages pre-contemplation (not ready), contemplation (getting ready), preparation (ready), action, and maintenance (Prochaska and Velicer, 1997) as a targeted factor, the enjoyment is a crude feeling. It is possible, that participants are feeling enjoyed and be motivated simultaneously, therefore it was adjusted for the motivation as a potential confounder.

### 2.5 Statistical methods

MVPA and bouted MVPA are described by mean and standard deviation for age groups and sex. Multivariate linear regression was used to examine the relationship between MVPA and bouted MVPA (dependent outcome variables) and the enjoyment of PA adjusted for age, sex, education, current smoking, and body mass index. The self-reported intrinsic motivation by the five stages of change of PA (Marcus et al., 1992) was measured to proof for a possibly confounding with enjoyment and was analysed descriptively. Metric variables were z-transformed. Analyses were performed using R 3.1.1 (The R Foundation for Statistical Computing). Participants with missing questionnaire data were excluded for the primary analysis. In a sensitivity analysis, patients' missing questionnaire entries were supplemented using multiple imputations with a median value imputation. A multiple imputation is a completion of missing data using existing data of similar patients' characteristics. For imputation of the missing questionnaire items, the R package ‘mice’ was used with 10 iterations.

## 3 Results

**Fig. 1:**
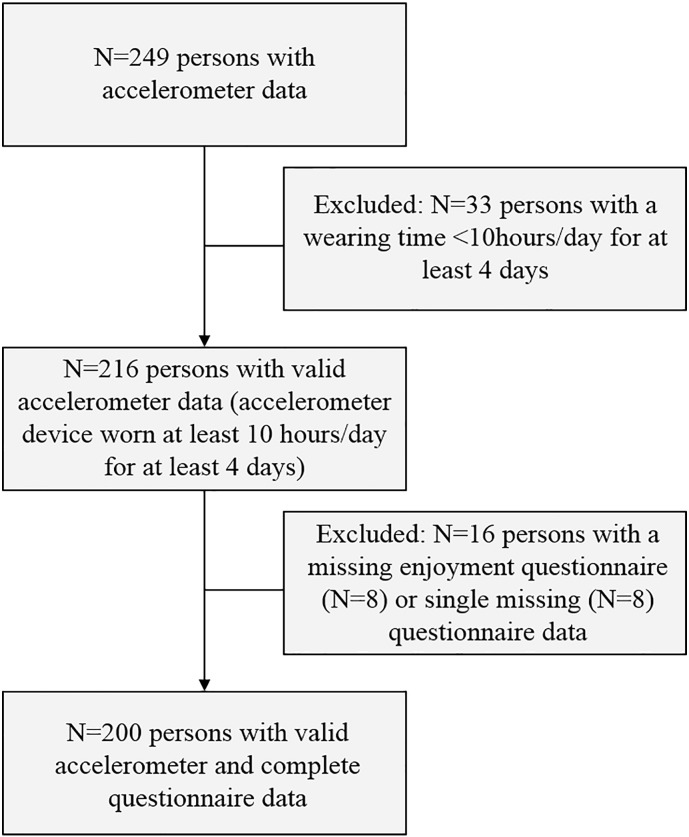
Flowchart for accelerometer and questionnaire data using the physical activity enjoyment scale (PACES) score.

The flow chart of the analysis is shown in Fig. 1 for existing accelerometer and questionnaire data. Single missing data describes one single missing questionnaire item instead of a missing enjoyment questionnaire.

The participants' characteristics at baseline are shown in Table 1.

**Table 1:** Participants' characteristics (N = 200 with complete accelerometer and questionnaire data).

Participants' characteristics	Men (n = 82)	Women (n = 118)	Total (n = 200)
Age (years, mean ± SD)	56.94 ± 10.04	55.92 ± 9.45	56.34 ± 9.69
Education (n, %) < 10 years 10 years ≥ 10 years	14 (17.1) 41 (50.0) 27 (32.9)	11 (9.3) 61 (51.7) 46 (40.0)	25 (12.5) 102 (51.0) 73 (36.5)
Occupation (n, %) Employed Unemployed	31 (37.8) 51 (62.2)	64 (54.2) 54 (45.8)	95 (47.5) 105 (52.5)
Recruitment setting (n, %) Health insurance General medical practices Job agencies	36 (43.9) 31 (37.8) 15 (18.3)	56 (47.5) 39 (33.1) 23 (19.5)	92 (46.0) 70 (35.0) 38 (19.0)
Body mass index (n, %) ≤ 25 kg/m^2^ 25–30 kg/m^2^ > 30 kg/m^2^	21 (25.6) 40 (48.8) 21 (25.6)	52 (44.1) 38 (32.2) 28 (23.7)	73 (36.5) 78 (39.0) 49 (24.5)
Smoking (n, %) Current smoking	20 (24.4)	23 (19.5)	43 (21.5)
Self-reported general health status (n, %) Excellent Very good Good Less good Bad	4 (4.9) 12 (14.6) 54 (65.9) 8 (9.8) 4 (4.9)	2 (1.7) 18 (15.3) 78 (66.1) 19 (16.1) 1 (0.8)	6 (3.0) 30 (15.0) 132 (66.0) 27 (13.5) 5 (2.5)
Intrinsic motivation (stages of change) Pre-contemplators (stage 1) Contemplators (stage 2) Preparers (stage 3) Actors (stage 4) Maintainers (stage 5)	16 (19.5) 5 (6.1) 9 (11.0) 3 (3.7) 49 (59.8)	7 (5.9) 20 (16.9) 10 (8.5) 9 (7.6) 72 (61.0)	23 (11.5) 25 (12.5) 19 (9.5) 12 (6.0) 121 (60.5)

SD: standard deviation.

In the cardiovascular examination program more women than men were included. Age, education and the proportion of the recruitment settings are similar between men and women. However, there are more male smokers (24.4% vs. 19.5%), more male participants without occupation (62.2%) and with pre-obesity (BMI 25–30 kg/m^2^: 48.8% vs. 32.2%) Slight differences exist in self-reported general health status (less good 9.8% vs. 16.1%), which represents gender-specific deviations in general health status and lifestyles. Most of all, the participants are in the highest stage of change for intrinsic motivation (60.5%).

The average time of light activity per day was 208.9 ± 56.9 (median 216) min/day for men and 228.9 ± 57.2 (median 220.0) for women. The average time in MVPA/day (including bouted MVPA) was 44.4 ± 27.3 (median 39, Table 2) min/day. MVPA decreased for higher aged study participants. Among participants > 45 years, the time of bouted MVPA was 8.1 ± 12.0 (median 4) min/day. All in all, 132 (66%) participants achieved bouted MVPA. Men spent on average 2 min more of bouted MVPA than women. Men in the age group 45–50 years showed the longest time of bouted MVPA (13.3 ± 17.5 min per day, Table 2).

**Table 2:** Moderate-to-vigorous physical activity (MVPA) and bouted MVPA in minutes/day by age and sex.

Age groups	Men		Women		Total	
	n	mean ± SD (median)	n	mean ± SD (median)	n	mean ± SD (median)
MVPA in minutes per day						
45–50 years	27	58.2 ± 34.8 (52)	40	49.8 ± 27.6 (43)	67	53.2 ± 30.7 (48)
51–60 years	22	45.2 ± 26.1 (38)	36	48.3 ± 25.0 (41)	58	47.1 ± 25.3 (41)
61–70 years	22	41.1 ± 27.3 (39)	30	31.0 ± 17.5 (30)	52	35.3 ± 22.4 (32)
71–75 years	11	38.3 ± 24.3 (36)	12	27.4 ± 18.8 (23)	23	32.6 ± 21.8 (29)
Total	82	47.4 ± 29.9 (42)	118	42.3 ± 25.2 (38)	200	44.4 ± 27.3 (39)

Bouted MVPA in minutes per day						
45–50 years	27	13.3 ± 17.5 (6)	40	6.9 ± 12.3 (3)	67	9.5 ± 14.9 (4)
51–60 years	22	8.1 ± 13.5 (2)	36	9.2 ± 11.7 (4)	58	8.8 ± 12.3 (4)
61–70 years	22	6.8 ± 7.9 (4)	30	6.6 ± 9.2 (4)	52	6.6 ± 8.6 (4)
71–75 years	11	7.4 ± 8.9 (4)	12	4.1 ± 6.1 (2)	23	5.7 ± 8.0 (3)
Total	82	9.4 ± 13.4 (5)	118	7.2 ± 10.9 (3)	200	8.1 ± 12.0 (4)

SD: standard deviation.

The average time in MVPA was for participants which were observed in spring 48.9 ± 37.0 (mean ± SD, n = 8), in summer 43.3 ± 25.0 (n = 37), in autumn 43.0 ± 26.8 (n = 115), and in winter 48.4 ± 29.0 (n = 40).

The self-reported PACES scores has a right-skewed distribution (99.99 ± 17.39, median = 104). The enjoyment of PA differs between male and female participants. In women, higher age is associated with higher enjoyment score, therefore, a stratified analysis was used to develop possible gender-specific effects. Men over 70 years show the lowest PACES scores (mean ± SD, 93.1 ± 18.2, see Table 3).

**Table 3:** Physical activity enjoyment scale by age and sex.

Physical activity enjoyment scale						
Age groups	Men		Women		Total	
	n	mean ± SD (median)	n	mean ± SD (median)	n	mean ± SD (median)
45–50 years	27	99.6 ± 13.3 (98)	40	95.5 ± 19.4 (97)	67	97.1 ± 17.2 (98)
51–60 years	22	95.3 ± 20.6 (100)	36	100.8 ± 19.9 (109)	58	98.7 ± 20.2 (107)
61–70 years	22	105.2 ± 13.0 (109)	30	104.8 ± 13.6 (108)	52	105.0 ± 13.3 (109)
71–75 years	11	93.1 ± 18.2 (101)	12	107.0 ± 13.9 (109)	23	100.4 ± 17.2 (104)
Total	82	99.1 ± 16.5 (101)	118	100.6 ± 18.1 (106)	200	100.0 ± 17.4 (104)

SD: standard deviation.

The results of the linear regression of square root transformed MVPA per day are shown in Table 4. The enjoyment of PA shows a significant positive association with MVPA/day (β = 0.18, 95% CI (0.05; 0.31), p = 0.009). Whereas age (β = − 0.33, 95% CI (− 0.48; − 0.18), p < 0.001) shows a significantly negative relationship with MVPA.

**Table 4:** Results of the linear regression for square root transformed moderate-to-vigorous physical activity (MVPA) in minutes/day (n = 200 participants) and the subgroup of participants with achieved bouted MVPA (n = 132).

	MVPA in minutes/day (n = 200)		Bouted MVPA in minutes/day (n = 132)^a^	
	Standardized β (95% confidence interval)	p-Value	Standardized β (95% confidence interval)	p-Value
Enjoyment (PACES)	0.18 (0.05; 0.31)	0.009	0.15 (− 0.02; 0.31)	0.082
Age (years)	− 0.33 (− 0.48; − 0.18)	< 0.001	− 0.06 (− 0.24; 0.12)	0.526
Sex (reference: male) Female	− 0.13 (− 0.26; 0.01)	0.062	− 0.12 (− 0.29; 0.04)	0.140
Education (reference: < 10 years) 10 years ≥ 10 years	0.09 (− 0.13; 0.31) 0.08 (− 0.14; 0.30)	0.406 0.478	0.01 (− 0.27; 0.29) 0.22 (− 0.05; 0.50)	0.937 0.119
Setting (reference: job agencies) Health insurance General medical practices	− 0.10 (− 0.31; 0.10) − 0.19 (− 0.38; 0.00)	0.330 0.060	− 0.52 (− 0.77; − 0.28) − 0.47 (− 0.70; − 0.23)	< 0.001 < 0.001
Body mass index (kg/m^2^)	0.01 (− 0.13; 0.15)	0.907	0.13 (− 0.04; 0.29)	0.138
Smoking (reference: smoker) Non-smoker	0.11 (− 0.04; 0.26)	0.159	0.12 (− 0.06; 0.31)	0.186

The model is adjusted for age, sex, education, recruitment setting, body mass index, and smoking; PACES: physical activity enjoyment scale.

a Participants with achieved bouted MVPA are analysed only.

In a sensitivity analysis, the regression was also performed with non-transformed MVPA, with similar results (results not shown).

For bouted MVPA and enjoyment of PA there is no significant relationship (β = 0.15, 95% CI (− 0.02; 0.31), p = 0.082). Subjects recruited at the health insurance and subjects recruited in general medical practices show significant reduced bouted MVPA (health insurance (β = − 0.52, 95% CI (− 0.77; − 0.28), p < 0.001); general medical practices (β = − 0.47, 95% CI (− 0.70; − 0.23), p < 0.001)) compared to participants recruited at their job agencies.

The regression results of the sensitivity analysis using median value imputation (n = 208) are similar to the results of the primary analysis (results not shown).

In Table 5 the results of the linear regression of MVPA/day stratified for sex are shown, whereas enjoyment of PA shows a significant positive association to MVPA for men (β = 0.34, 95% CI (0.13; 0.55), p = 0.002), but not for women (β = 0.09, 95% CI (− 0.09; 0.26), p = 0.347).

**Table 5:** Results of the linear regression for square root transformed moderate-to-vigorous physical activity (MVPA) in minutes/day stratified by sex.

	MVPA in minutes/day for men (n = 82)		MVPA in minutes/day for women (n = 118)	
	Standardized β (95% confidence interval)	p-Value	Standardized β (95% confidence interval)	p-Value
Enjoyment (PACES)	0.34 (0.13; 0.55)	0.002	0.09 (− 0.09; 0.26)	0.347
Age (years)	− 0.22 (− 0.49; 0.04)	0.099	− 0.41 (− 0.61; − 0.22)	< 0.001
Education (reference: < 10 years)				
10 years	0.09 (− 0.24; 0.42)	0.591	0.07 (− 0.25; 0.39)	0.665
≥ 10 years	0.09 (− 0.22; 0.39)	0.588	0.05 (− 0.28; 0.38)	0.769
Setting (reference: job agencies)				
Health insurance	− 0.11 (− 0.47; 0.25)	0.560	− 0.12 (− 0.37; 0.14)	0.366
General medical practices	− 0.27 (− 0.60; 0.07)	0.126	− 0.15 (− 0.39; 0.09)	0.217
Body mass index (kg/m^2^)	0.11 (− 0.10; 0.33)	0.312	− 0.08 (− 0.26; 0.10)	0.399
Smoking (reference: smoker)				
Non-smoker	0.06 (− 0.19; 0.31)	0.631	0.15 (− 0.04; 0.34)	0.133

The model is adjusted for age, sex, education, recruitment setting, body mass index, and smoking; PACES: physical activity enjoyment scale.

## 4 Discussion

We analysed the objectively measured PA of adults aged 40–75 years without a history of cardiovascular diseases. The percentage of MVPA was higher than for bouted MVPA. Self-reported enjoyment of PA was a positive determinant of higher MVPA per day but not for bouted MVPA. The positive association of enjoyment of PA to MVPA may imply that positive feelings regarding PA could increase adults' sporadic MVPA per day. Enjoyment of PA can also be a positive secondary effect of receiving physical fitness, especially for sporadic MVPA. Older adults showed less time in MVPA per day, similar as reported by Johannsen et al. (2008) as well as Hagberg and colleagues (Hagberg et al., 2009). Although, in the stratified approach, we found no association of enjoyment to MVPA for women. In non-showed results, we adjusted for this randomization group selection as published in van den Berg et al. (2017), but there was no effect.

### 4.1 Summary

A negative relationship was observed for participants who were recruited in general medical practices and by the health insurance company compared to participants recruited in job agencies. This could be associated with higher age in the settings compared to the persons recruited at the job agency. For the other covariates as education, body mass index, and current smoking status we find no relationship to objectively measured PA in our multivariate regression approach. The association between higher enjoyment and higher activity level measured by the long version of the International Physical Activity Questionnaire (Craig et al., 2003) as in our present study is similar to the results described by Santos et al., where women's intrinsic motivation was associated with leisure-time PA (β = 0.67, 99% CI (0.19; 1.15), (Santos et al., 2016)). In contrast to this approach which was based on self-reports we find an association with objectively measured PA.

A reason for the higher percentage of sporadic than bouted MVPA could be that sporadic MVPA is easier to integrate in every-day-life. Robson et al. found that MVPA did not need performed in 10 min bouts (Robson and Janssen, 2015). The result that enjoyment of PA seems to have no effect on bouted MVPA could indicate that for participants with achieved bouted MVPA the enjoyment of PA cannot increase the time of being physical active, similar to results by Bond et al. (2016). A reason of no relation could be that there is a saturation effect for the need of being physical active. For bouted MVPA there seems to be more (unobserved) factors to be physical active than enjoyment of PA only. Due to modern lifestyle behaviours and sedentary office occupations, especially the leisure time should be used to be more physically active for middle-aged adults. Although, the proportion of time in bouted MVPA was low for the total study population which means that the subjects were more sporadically physically active.

Finally, in the stratified analysis by sex it was shown that for men the enjoyment has a significant relationship, whereas for women there is no association.

A possible explanation for this sex-specific differences could be that in women, the association between PA and other factors is more complex. Dukanovic found that psychological-social reasons as social contacts and community spirit during be physical active are more important for women's health than physiological reasons (Dukanovic et al., 2015).

In a systematic review by Trost et al. the positive associations for individual, social, and environmental factors to overall PA in adults were analysed, enjoyment of exercise shows positive associations in two studies (Booth et al., 2000, Leslie et al., 1999).

### 4.2 Strengths

The activity level of 200 participants was objectively measured via accelerometer device. The influence of the self-reported enjoyment on both, general MVPA and bouted MVPA was considered. The physical activity enjoyment score is a validated instrument with simple handling and a useful tool to perceive subjects' feelings of PA. Further, a possible confounding to intrinsic motivation was taken into account (results not shown).

### 4.3 Limitations

A limitation of this approach was the cross-sectional design which cannot show long-term effects. Further, the explanation of the variance of the regression model was rather poor, which implied that further factors influence the time spent in MVPA per day. In a systematic approach socioeconomic status and perceived self-efficacy showed the strongest association to PA (Trost et al., 2002). There could also be a mediated factor for enjoyment and self-efficacy as shown by Lewis et al. (2016), which could not be evaluated here. Possible further factors could be environmental aspects (for example, distance to parks or swimming pools), social (individual training-level or access and opportunity to join exercise courses) or other aspects that were not regarded here. Further, weather-related, outdoor-temperature or individual weather-dependent deviations (every-day-runner vs. fair-weather-runner) were not regarded in our study approach which could be have an influence on bouted MVPA per day. The season of the year and the presence of an occupation showed no significant difference of participants' behaviour to be physical active (results not shown).

There is a restriction for the validity of accelerometer measurements for some activities (e.g. for biking and swimming) for the sample frequency (Brond and Arvidsson, 2016) used non-wear algorithm, cut points, and epoch length (Logan et al., 2016).

Finally, the participants' individual training-level, the proportion of exercise (running or training in fitness studio) and daily-life PA (activity in garden or household) could not be considered in this approach. In addition, enjoyment of PA was a self-report to measure the individual perception of being physical active. But enjoyment of PA could be useful in prevention settings and even for long-term behaviour change (McArthur and Raedeke, 2009).

## 5 Conclusion

PA is an essential factor of prevention programs in middle-aged subjects. Enjoyment of PA could be an important motivational factor to become more physical active and to enhance the individual PA level. For bouted MVPA self-reported enjoyment cannot reach an enhancement of PA. A higher enjoyment of PA can help to be physically active in both occupational and leisure time in everyday life. Therefore, enjoyment should be considered a target among other motivational factors in preventive (gender-specific) physical activity studies.

## Conflict of interest

The authors declare that there is no conflict of interest.

## Source of funding

This work was supported by the German Centre for Cardiovascular Research (DZHK, grant number 81Z7400174) and the German Federal Ministry of Education and Research.

## References

[bb0005] Aldana S.G., Sutton L.D., Jacobson B.H., Quirk M.G. (1996). Relationships between leisure time physical activity and perceived stress. Percept. Mot. Skills.

[bb0015] Bize R., Johnson J.A., Plotnikoff R.C. (2007). Physical activity level and health-related quality of life in the general adult population: a systematic review. Prev. Med..

[bb0020] Blair S.N., Kohl H.W., Paffenbarger R.S., Clark D.G., Cooper K.H., Gibbons L.W. (1989). Physical fitness and all-cause mortality. A prospective study of healthy men and women. JAMA.

[bb0025] Bond D.S., Graham Thomas J., Vithiananthan S. (2016). Changes in enjoyment, self-efficacy, and motivation during a randomized trial to promote habitual physical activity adoption in bariatric surgery patients. Surg. Obes. Relat. Dis..

[bb0030] Booth M.L., Owen N., Bauman A., Clavisi O., Leslie E. (2000). Social-cognitive and perceived environment influences associated with physical activity in older Australians. Prev. Med..

[bb0035] Brond J.C., Arvidsson D. (2016). Sampling frequency affects the processing of Actigraph raw acceleration data to activity counts. J. Appl. Physiol..

[bb0040] Caspersen C.J., Powell K.E., Christenson G.M. (1985). Physical activity, exercise, and physical fitness: definitions and distinctions for health-related research. Public Health Rep..

[bb0045] Cleland V., Squibb K., Stephens L. (2017). Effectiveness of interventions to promote physical activity and/or decrease sedentary behaviour among rural adults: a systematic review and meta-analysis. Obes. Rev..

[bb0050] Craig C.L., Marshall A.L., Sjostrom M. (2003). International physical activity questionnaire: 12-country reliability and validity. Med. Sci. Sports Exerc..

[bb0055] Dukanovic N., Masic Z., Kostovski Z., Siric V., Blazevic S. (2015). Physical activity as a function of women's health. Coll. Antropol..

[bb0060] Ekelund U., Ward H.A., Norat T. (2015). Physical activity and all-cause mortality across levels of overall and abdominal adiposity in European men and women: the European Prospective Investigation into Cancer and Nutrition Study (EPIC). Am. J. Clin. Nutr..

[bb0065] Elavsky S., McAuley E., Motl R.W. (2005). Physical activity enhances long-term quality of life in older adults: efficacy, esteem, and affective influences. Ann. Behav. Med..

[bb0070] Gill D.L., Hammond C.C., Reifsteck E.J. (2013). Physical activity and quality of life. J. Prev. Med. Public Health.

[bb0075] Gretebeck R.J., Montoye H.J. (1992). Variability of some objective measures of physical activity. Med. Sci. Sports Exerc..

[bb0080] Hagberg L.A., Lindahl B., Nyberg L., Hellenius M.L. (2009). Importance of enjoyment when promoting physical exercise. Scand. J. Med. Sci. Sports.

[bb0085] Haskell W.L., Nelson M.E. (2008). Physical Activity Guidelines Advisory Committee Report, 2008. To the Secretary of Health and Human Services.

[bb0090] Haskell W.L., Lee I.M., Pate R.R. (2007). Physical activity and public health: updated recommendation for adults from the American College of Sports Medicine and the American Heart Association. Circulation.

[bb0095] Hu F.B., Sigal R.J., Rich-Edwards J.W. (1999). Walking compared with vigorous physical activity and risk of type 2 diabetes in women: a prospective study. JAMA.

[bb0100] Inoue S., Ohya Y., Odagiri Y. (2011). Sociodemographic determinants of pedometer-determined physical activity among Japanese adults. Am. J. Prev. Med..

[bb0105] Jekauc D., Voelkle M., Wagner M.O., Mewes N., Woll A. (2013). Reliability, validity, and measurement invariance of the German version of the physical activity enjoyment scale. J. Pediatr. Psychol..

[bb0110] Johannsen D.L., DeLany J.P., Frisard M.I. (2008). Physical activity in aging: comparison among young, aged, and nonagenarian individuals. J. Appl. Physiol..

[bb0115] Katzmarzyk P.T., Gledhill N., Shephard R.J. (2000). The economic burden of physical inactivity in Canada. CMAJ.

[bb0120] Kendzierski D., DeCarlo K.L. (1991). Physical activity enjoyment scale: two validation studies. J. Sport Exerc. Psychol..

[bb0125] Kriska A.M., Saremi A., Hanson R.L. (2003). Physical activity, obesity, and the incidence of type 2 diabetes in a high-risk population. Am. J. Epidemiol..

[bb0130] Lee I.M., Shiroma E.J., Lobelo F. (2012). Effect of physical inactivity on major non-communicable diseases worldwide: an analysis of burden of disease and life expectancy. Lancet.

[bb0135] Leslie E., Owen N., Salmon J., Bauman A., Sallis J.F., Lo S.K. (1999). Insufficiently active Australian college students: perceived personal, social, and environmental influences. Prev. Med..

[bb0140] Lewis B.A., Williams D.M., Frayeh A., Marcus B.H. (2016). Self-efficacy versus perceived enjoyment as predictors of physical activity behaviour. Psychol. Health.

[bb0145] Li J., Siegrist J. (2012). Physical activity and risk of cardiovascular disease—a meta-analysis of prospective cohort studies. Int. J. Environ. Res. Public Health.

[bb0150] Logan G.R., Duncan S., Harris N.K., Hinckson E.A., Schofield G. (2016). Adolescent physical activity levels: discrepancies with accelerometer data analysis. J. Sports Sci..

[bb0155] Loprinzi P.D., Davis R.E. (2016). Bouted and non-bouted moderate-to-vigorous physical activity with health-related quality of life. Prev. Med. Rep..

[bb0160] Lozano R., Naghavi M., Foreman K. (2012). Global and regional mortality from 235 causes of death for 20 age groups in 1990 and 2010: a systematic analysis for the Global Burden of Disease Study 2010. Lancet.

[bb0165] Marcus B.H., Rossi J.S., Selbi V.C., Niaura R.S., Abrams D.B. (1992). The stages and processes of exercise adoption and maintenance in a worksite sample. Health Psychol..

[bb0170] McArthur L.H., Raedeke T.D. (2009). Race and sex differences in college student physical activity correlates. Am. J. Health Behav..

[bb0175] Mensink G., Robert-Koch-Institut (2003). Beiträge zur Gesundheitsberichterstattung des Bundes - Bundes-Gesundheitssurvey: Körperliche Aktivität - Aktive Freizeitgestaltung in Deutschland.

[bb0180] Mullen S.P., Olson E.A., Phillips S.M. (2011). Measuring enjoyment of physical activity in older adults: invariance of the physical activity enjoyment scale (paces) across groups and time. Int. J. Behav. Nutr. Phys. Act..

[bb0185] Murphy M.H., Blair S.N., Murtagh E.M. (2009). Accumulated versus continuous exercise for health benefit: a review of empirical studies. Sports Med..

[bb0190] Prince S.A., Adamo K.B., Hamel M.E., Hardt J., Connor G.S., Tremblay M. (2008). A comparison of direct versus self-report measures for assessing physical activity in adults: a systematic review. Int. J. Behav. Nutr. Phys. Act..

[bb0195] Prochaska J.O., Velicer W.F. (1997). The transtheoretical model of health behavior change. Am. J. Health Promot..

[bb0200] Rejeski W.J., Mihalko S.L. (2001). Physical activity and quality of life in older adults. J. Gerontol. A Biol. Sci. Med. Sci..

[bb0205] Robson J., Janssen I. (2015). Intensity of bouted and sporadic physical activity and the metabolic syndrome in adults. PeerJ.

[bb0210] Roth M.A., Mindell J.S. (2013). Who provides accelerometry data? Correlates of adherence to wearing an accelerometry motion sensor: the 2008 Health Survey for England. J. Phys. Act. Health.

[bb0215] Sallis J.F., Johnson M.F., Calfas K.J., Caparosa S., Nichols J.F. (1997). Assessing perceived physical environmental variables that may influence physical activity. Res. Q. Exerc. Sport.

[bb0220] Santos I., Ball K., Crawford D., Teixeira P.J. (2016). Motivation and barriers for leisure-time physical activity in socioeconomically disadvantaged women. PLoS One.

[bb0225] Sasaki J.E., John D., Freedson P.S. (2011). Validation and comparison of ActiGraph activity monitors. J. Sci. Med. Sport.

[bb0230] Schneider M., Schmalbach P., Godkin S. (2017). Impact of a personalized versus moderate-intensity exercise prescription: a randomized controlled trial. J. Behav. Med..

[bb0235] Stattin K., Michaelsson K., Larsson S.C., Wolk A., Byberg L. (2017). Leisure-time physical activity and risk of fracture: a cohort study of 66,940 men and women. J. Bone Miner. Res..

[bb0240] Strath S.J., Kaminsky L.A., Ainsworth B.E. (2013). Guide to the assessment of physical activity: clinical and research applications: a scientific statement from the American Heart Association. Circulation.

[bb0245] Strawbridge W.J., Deleger S., Roberts R.E., Kaplan G.A. (2002). Physical activity reduces the risk of subsequent depression for older adults. Am. J. Epidemiol..

[bb0250] Tremblay M.S., Warburton D.E., Janssen I. (2011). New Canadian physical activity guidelines. Appl. Physiol. Nutr. Metab..

[bb0255] Troiano R.P., Berrigan D., Dodd K.W., Masse L.C., Tilert T., McDowell M. (2008). Physical activity in the United States measured by accelerometer. Med. Sci. Sports Exerc..

[bb0260] Trost S.G., Owen N., Bauman A.E., Sallis J.F., Brown W. (2002). Correlates of adults' participation in physical activity: review and update. Med. Sci. Sports Exerc..

[bb0010] van den Berg N., Ulbricht S., Schwaneberg T. (2017). The influence of wearing schemes and supportive telephone calls on adherence in accelerometry measurement: results of a randomized controlled trial. Patient Prefer. Adherence.

[bb0265] Westerterp K.R. (1999). Physical activity assessment with accelerometers. Int. J. Obes. Relat. Metab. Disord..

[bb0270] Weymar F., Braatz J., Guertler D., van den Berg N., Meyer C. (2015). Characteristics associated with non-participation in 7-day accelerometry. Prev. Med. Rep..

[bb0275] WHO (2016). Physical Activity and Adults - Recommended Levels of Physical Activity for Adults Aged 18–64 Years.

